# New onset delusional infestation following abrupt cessation of hydroxyzine

**DOI:** 10.1016/j.jdcr.2023.06.042

**Published:** 2023-07-08

**Authors:** Rose Velasco, Jesse J. Keller

**Affiliations:** a University of Illinois Chicago, College of Medicine, Chicago, Illinois; b Oregon Health & Science University, Department of Dermatology, Portland, Oregon

**Keywords:** delusional infestation, dermatology, hydroxyzine, psychodermatology

## Introduction

Delusional infestation (DI) is a psychocutaneous condition in which patients exhibit a fixed, false belief that they are infested with an insect or a parasite despite evidence indicating the contrary. In this article, we present a case of DI presenting after the abrupt discontinuation of long-term use of the H1 antihistamine hydroxyzine.

## Case report

A 59-year-old man with a history of mild psoriasis, long smoking history, obesity, and hepatitis C status after medical treatment was closely followed by the dermatology department for over 2 years for repeated hospitalizations for erythroderma and new onset of 10/10 pruritic palmoplantar rash. The differential diagnosis included psoriasis, atopic dermatitis, allergic contact dermatitis, and acrodermatitis paraneoplastica (Bazex syndrome), for which a CT scan of the chest was obtained, which did not show any evidence of malignancy. Two cutaneous biopsies were performed, which showed a mixed histologic picture of psoriatic spongiotic dermatitis. He responded well to cyclosporine, although he developed acute decreased renal function each of the three times cyclosporine was utilized. Long-term prednisone treatment was complicated by pathologic rib fracture. He failed numerous therapies, including dupilumab (2 months), adalimumab (5 months), and guselkumab (7 months), in conjunction with longitudinal clobetasol and acitretin. He rapidly recovered during each hospitalization but quickly flared again every time after he was discharged from the hospital, raising the question of poor compliance and/or an allergic contact dermatitis. Infliximab was started to better assess compliance, and the patient improved to the point of successfully tapering prednisone, although his rash continued to persist. During a subsequent hospitalization, his eruption evolved to include newly noted, sharp demarcation across the dorsal feet, correlating with the shape of his footwear. We suspected allergic contact dermatitis to footwear and referred him for patch testing. During his telephonic screening, he was instructed to discontinue cyclosporine 2 weeks prior to patch placement, but due to a misunderstanding, he also stopped acitretin and nightly hydroxyzine for nocturnal pruritus. When he visited for patch testing, he reported a new concern of “spiders” on and under his skin, pointing to inorganic debris on his clothing and legs, which raised concerns for delusional parasitosis. He was advised to restart only hydroxyzine and educated regarding strategies to avoid allergens in shoe rubber and adhesives, for which he tested positive on patch testing. Within 1 month, his palms and soles remained inflamed as he had not yet made modifications to his footwear. He did not spontaneously mention infestation. When prompted about the insect infestation, he stated that the condition had completely resolved.

## Discussion

We concluded that this presentation of DI was likely caused by hydroxyzine withdrawal. Using the Naranjo Adverse Drug Reaction Probability Scale, he earned a total of 4 points as follows: 3 points for temporal nature of symptoms correlating with drug withdrawal and reintroduction, 1 point for previous reports of sedative withdrawal correlating with DI, and another 1 point for objective evidence of the adverse event (matchbox sign); however, he lost 1 point for the possibility that DI could be due to another cause, such as unsuspected illicit drug use. Historically, this patient has never reported alcohol use and throughout our follow-ups over the years, we neither suspected illicit drug use nor had a reason to perform drug screening, although a negative screen would have been ideal. Another potential confounding factor was that the patient’s sister had passed away just weeks before, and psychologic stress could have contributed to his acute presentation of DI, but it is unlikely for this to abate in such a rapid fashion.

DI may be caused by stimulating medications or substances (amphetamines and cocaine)^1^ or withdrawal of sedative medications or substances (opioids, benzodiazepines, and alcohol).^2^, ^3^, ^4^ One case report describes new-onset DI following acute withdrawal from the pharmacologic sedative trazodone.^5^ To our knowledge, we report the first known cases of DI caused by cessation of hydroxyzine, a histamine-1 receptor (H1R) antagonist. Hydroxyzine and other first-generation antihistamines are able to cross the blood-brain barrier and target H1Rs throughout the brain, leading to sedation, an often desired side effect in the treatment of insomnia.^6^ This also makes hydroxyzine an ideal drug choice in dermatology, when treating intractable nocturnal pruritus, such as in our patient, and it has become a popularly prescribed medication throughout the field for a number of other conditions.^7^ In addition to targeting H1R, hydroxyzine also blocks muscarinic, serotonergic, and dopaminergic receptors.^8^^,^^9^ We hypothesize that rapid withdrawal of this broad antagonism may lead to acute stimulation of the nervous system. In addition, multiple dopamine agonists have been theorized to cause activation of the mesolimbic dopaminergic pathway, leading to DI.^1^^,^^10^ Thus, withdrawal of hydroxyzine’s dopaminergic blockade could also contribute to this presentation.

As hydroxyzine is a frequently prescribed medication for pruritus, dermatologists should be aware of the possibility of developing DI without prudent tapering. Dermatologists are also encouraged to preferentially utilize second generation H1 antihistamines (cetirizine, fexofenadine, and loratadine) for their more benign side-effect profile.

## Conflicts of interest

None disclosed.

## References

[1] Bramness J.G., Rognli E.B. (2016). Psychosis Induced by Amphetamines. Curr Opin Psychiatry.

[2] Mowla A., Asadipooya K. (2009). Delusional Parasitosis Following Heroin Withdrawal: A Case Report. Am J Addict.

[3] Lepping P., Noorthoorn E.O., Kemperman P.M.J.H. (2017). An International Study of the Prevalence of Substance use in Patients with Delusional Infestation. J Am Acad Dermatol.

[4] Knapp B., Tito E., Espiridion E.D. (2019). Delusional Parasitosis in a Patient with Alcohol-induced Psychotic Disorder. Cureus.

[5] Peabody C.A. (1987). Trazodone Withdrawal and Formication. J Clin Psychiatry.

[6] Tashiro M., Mochizuki H., Iwabuchi K. (2002). Roles of Histamine in Regulation of Arousal and Cognition: Functional Neuroimaging of Histamine H1 Receptors in Human Brain. Life Sci.

[7] Hsieh C.Y., Tsai T.F. (2021). Use of H-1 Antihistamine in Dermatology: More than Itch and Urticaria Control: A Systematic Review. Dermatol Ther (Heidelb).

[8] Burgazli C.R., Rana K.B., Brown J.N., Tillman F. (2023). Efficacy and Safety of Hydroxyzine for Sleep in Adults: Systematic Review. Hum Psychopharmacol.

[9] Haraguchi K., Ito K., Kotaki H., Sawada Y., Iga T. (1997). Prediction of Drug-Induced Catalepsy Based on Dopamine D1, D2, and Muscarinic Acetylcholine Receptor Occupancies. Drug Metab Dispos.

[10] Kemperman P.M.J.H., Bruijn T.V.M., Vulink N.C.C., Mulder M.M.C. (2022). Drug-Induced Delusional Infestation. Acta Derm Venereol.

